# Dr. Goddard’s Address

**Published:** 1849-10

**Authors:** 


### DENTAL REGISTER OF THE WEST.

VOL. III. OCTOBER, 1849. NO. 1.

### DR. GODDARD'S ADDRESS,

DELIVERED BEFORE THE MISSISSIPPI VALLEY ASSOCIATION OF DENTAL SURGEONS, AT THEIR FIFTH ANNUAL MEETING, HELD IN LOUISVILLE, KY., SECOND TUESDAY OF SEPTEMBER, 1849.

Gentlemen: In availing myself of the honor which has devolved upon me, of addressing you on the present occasion as your President, I feel very sensibly the necessity of invoking, at the outset, your indulgent consideration of my humble efforts to merit your attention.

I cannot but feel great diffidence in my own ability to meet your reasonable expectations of doing justice to any subject connected with our profession, when I see around me associates so distinguished in the fields of literature and science. Indeed this diffidence would be insurmountable, if I were not emboldened by calling to mind the harmonious relations which have hitherto subsisted between us. Under the influence of this pleasing and soothing reflection, I feel reassured that I am addressing friends more disposed to encourage than to criticise efforts designed for the promotion of mutual intercourse of sentiment and co-operation, and not for the display of mere personal pretensions.

At this re-union, we should doubtless have had reason, mutually, to congratulate ourselves on our professional career, since our last meeting, had we not felt the effects of that general suspension of business which has been the result of the

WI
DE346G

presence of Cholera in the Mississippi Valley. It is one of the characteristics of this withering scourge, that it paralyses communities as well as individuals. Happily, unlike old-world communities, previously worn out, or in decay from social or political disease, ours possesses a youthful vitality so elastic, that, like the bounding spring, temporarily overweighed, the moment the extreme pressure is removed, it regains its wonted position, and its play is restored, justifying our renewed confidence in its durability, having passed the ordeal of its strength.

It is gratifying to know that we are again free from this baleful and blighting pestilence: doubly gratifying to see that the recuperative energies of our community are fast recalling the hum of business in our midst, and the cheering evidences of successful industry every where around us. Such resuscitated animation must speedily efface every gloomy shadow of the past. It will of course be understood, that I say this in a collective sense, with reference to the general prosperity of the community. But in that community, I regret to say, many a vacant seat on the social hearth will be found, many a shaded brow, the gloom of which cannot be dissipated by the sunshine of worldly prosperity. The great mass of national vitality, regardless of these, rolls on its mighty wave, overwhelming the individual grief which stoops to weep, and "will not be comforted." To this mass of vitality, we, as a professional association, belong. To share in the onward movement and its results, we too, must resuscitate our energies, and advance hopefully to the participation. If, for a season, we have shared in the general depression, we may also expect to share in the more lasting recuperation. Each profession is but a member of the body politic; yet so identical is the vitality of all the members, that whatever of good or evil affects the heart, affects the whole, through every artery, vein and nerve. And here, it seems not inappropriate that I should submit to you some views in relation to our national body politic, in connexion with its professional members, which, possibly, have escaped general consideration. If there is any thing in them of value, you will appreciate it; but if, on the other hand, my "notions" seem to you crude or unimportant, you will, I have no doubt, at least receive them with the indulgent belief that they have originated in a very pardonable national vanity.

Gentlemen: I am one of those—and I believe their name is now Legion in this broad Union, who conceive that the United States is destined, by the Great Ruler of human events, to be at once the example, the arbiter, and the Christian civilizer of less fortunate nations of the earth. What is the first principle of this great power? It is the individual sovereignty of our people, guided by knowledge, and sustained by a co-operative energy, unequalled in the history of the world. These are things of which no probable changes can deprive us, for they are self generating. They are fenced round and secured by political institutions, all but God-like in their conception, if not superhuman in the far-seeing magnitude of their design. To bring the application of these remarks at once to ourselves, I will here observe that the glorious institutions of our happy land, are admirably constituted to encourage and ennoble every useful branch of practical science. It is only by contrasting effects under them, with those under political institutions of a different character, that we can rightly appreciate the blessings of the superiority which we enjoy. I may here very reasonably select for example the condition and prospects of our own profession at the present moment in the United States, and inquire how is it probable it would compare with the same profession in the most favored nations of the old world. Without the data to reason from facts, we may approximate the same results, in reasoning from analogies and causes which are inseparable from their consequences. We find in the United States, the profession of Dental Surgery now standing side by side with the professions of Surgery and Medicine,—co-operating with them in scientific fellowship. It is no longer denied its distinct and equal rank. It boasts of members second to those of no other profession, in mental capacity, literary attainments, and scientific skill. It is as fully and as extensively patronized by the community, as its co-ordinate professions. This extent of encouragement is due to many peculiar elements in the composition of our national society. Our people are a sovereign people in the true literal sense of the term ; governing themselves for their own advantage, and promoting individual prosperity by identifying it with national prosperity. Hence, in our community there is a universal diffusion of sufficiency of means to insure social comforts, if not luxuries; wide-spread knowledge, general education; civilization always progressive ; and the highest appreciation of human refinement.

No other country in the world can boast of such a population. We have no *canaille*, like the continental nations of Europe; no *lazaroni*—no brute ignorance leaving the human form a mere laboring machine—no starving paupers—no senseless mobs, incapable of distinguishing or understanding the good or evil of political institutions. What in other countries is but a very limited portion of their communities, the educated middle ranks, standing between the aristocracy and the laboring classes, will even contrast unfavorably with our whole population; because we know of no superiors in rank, and possess a moral energy, too elevated to be damped, restrained, or over-shadowed by aristocratic assumptions. Whilst France and England can have but a few hundred thousands of their middle classes worthy of comparison with our citizens, we have our twenty millions, equal to their intelligent classes.

The field of patronage in those countries, for a profession like ours, is limited to the upper and middle ranks; but becomes utterly barren when extended to their mass of population. Here it is altogether different. Our field of patronage is unlimited, embracing our whole population ; because we have no rank that does not appreciate the refinements and enjoyments of life, which render our services desirable, if not necessary.

Yes, gentlemen, it is no unfounded assumption to maintain that the people of the United States are a free, independent and enlightened people, such as no other nation in the world can boast of. They are collectively better educated, better fed, better clothed, and farther removed from the grinding cares of poverty, than the people of any other country on the face of the globe. They are consequently in a social position to aspire to refinement, to appreciate personal and domestic comforts, and to promote the elegancies of life which contribute most to social enjoyment. We can hardly, ourselves, appreciate this bright and distinguishing phase of our nationality; partly because we cease to reason upon things familiar and indisputable, and partly because, in a general sense, we have no opportunity of witnessing the contrast which the domestic condition of foreign countries presents. But occasionally, we have the graphic testimony of foreigners themselves when they sojourn for a season among us. Allow me to read an extract or two from a letter addressed by the great Apostle of Temperance to the Mayor of the city of New York, in July last:

"From the moment I caught the first glimpse of American land, every incident has awakened renewed pleasure and delight —I have gazed with rapture on the bold outline of your coast, and have admired the beautiful scenery of your noble bay, unrivaled for its maritime capabilities, and designed by nature as the great entrêpot of trade and of commerce for the Western world. I have seen your majestic rivers, dotted with richly freighted vessels, bearing the teeming produce of your luxuriant soil to far distant nations; and Oh! sir, I could not look on those winged messengers of peace and plenty, without associating with them the magnanimous bounty of a brave people to an afflicted nation.

I have visited your busy warehouses, your thronged streets and bustling thoroughfares, and have been forcibly struck with those external evidences of mercantile greatness and prosperity, which shadow forth the high commercial destiny that yet awaits your already glorious republic. I have seen in the comfort and abundance enjoyed by all, in the total absence of squalid poverty, and in the liberal remuneration which awaits honest toil, proofs of prosperity, which contrast strikingly with scenes that have often harrowed my soul in that poor old country, which, trodden down and oppressed as she is, is still the land of my birth and of my affections. I have visited your god-like institutions, upheld with a munificence worthy of your mighty republic, in which you imitate at an humble distance, the mercy of the Redeemer, making "the blind to see, the dumb to speak." I have minutely inspected their internal arrangements, and witnessed, with intense satisfaction, the philanthropic system and the absence of all religious exclusion on which those asylums, sacred to humanity, are based and conducted. I fervently pray that, "He who holds in his hands the destinies of nations," may make yours worthy of the favors He has bestowed; and with pure hearts, pure hands, and sleepless vigilance, that you may guard and defend, to the end of time, the great charge he has committed to your keeping."

In a country, thus truly depicted, of unrivaled national prosperity; in a community sharing so largely and so universally in this prosperity; it is obvious, that every branch of scientific utility must necessarily be exclusively encouraged. The constant tendency of accumulating means is to stimulate taste for enjoyment, and to excite aspirations for refinement of mind and habits. This taste is discernible in elegance or appropriateness of dress, in amenity of manners, in the care of personal appearances, and in the harmony of social intercourse. With these tendencies, the aid of the Dental Surgeon must necessarily be extensively invoked. The want of that aid is felt by those in health as well as by those afflicted with disease. A universal feeling becomes awakened that care is due to that mechanism by which the vital process of mastication is performed, and by which the precision of articulation in the intercourse of mind with mind, is preserved or improved. This care becomes an indispensable object of personal comfort if not enjoyment. The sentiment of its advantages becomes widely diffused in refined communities, until, at length, the encouragement it offers, enlists in the profession the solicitude of genius, and the ambition of men gifted with the highest mental attainments. I trust I have said enough to show the natural process by which our profession has been fostered and encouraged in the United States, until it has reached its present eminence.

Reverting to the history of other countries and other times, we do not find that the records of antiquity furnish any proof that the Egyptians, Greeks, or Romans, (with whose domestic civilization we are best acquainted,) considered Dental Surgery a distinct profession. The probability seems to be that Dentistry was considered separately with reference to the mere mechanical operation of extraction of teeth, and the treatment of pain proceeding from dental affections; one, perhaps attended to by an operator merely distinguished for adroitness, and the other to the medical man most esteemed for his successful treatment of local ailments. There is so little data for further conjecture in reference to antiquity, that we may abandon that field of inquiry, and come down to the middle ages where the refinements of civilization pervaded the western confines of Europe. And here we find no evidence of any considerable advance in the profession of dentistry. Even in countries far advanced in civilization, the nostrums of empirics and mountebanks, seem to have been more universally esteemed and encouraged, than the science of Dentistry as a distinct profession. Coming down to the golden age of literature in France and England, the reign of Louis XIV in the former, and of Queen Anne in the latter, we find that the professions of Barber and Surgeon were considered legitimately united, the prevailing opinion seeming to be, that the best performer of the tonsure must necessarily be the best extractor of the teeth, and the best phlebotomist.

Why, indeed, there should have been such an association of ideas, it is difficult to conjecture, unless it may be assumed that the scissors for cutting the hair were so conveniently contrived as to make one of the blades the best of lancets, and the other the best lever for prying out a tooth; or, it may have been, that the barbers who shaved and washed the elders had the largest basins for catching the superfluous blood of the youngsters, and the best chairs for seating the victims of tooth-ache during the manipulation of *extraction*, no doubt too often leading to *distraction*. Be that as it may, we do not find that Dentistry as a regular profession, had established itself respectably among our ancestors till the 18th century. Indeed, as a totally distinct profession, it can hardly be regarded clearly established in England, till within the first quarter of the present century. Its advancement since, both in the old world and the new, has been rapidly progressive. Perhaps of late years this advance has been more conspicuous and extensive in the United States, for reasons already assigned, than in any other country. And it is now my conviction that we must, aided by our system of association and intercommunication, take the lead of our profession in Europe. Neither England, France, or any other of the continental countries of Europe, can, from the nature of circumstances, admit of the same degree of encouragement to the profession which is afforded in this country. The condition of their populations is not favorable to it. Only very limited portions of their populations can afford to support, much less to encourage, a distinct profession not immediately felt to be necessary to the preservation of life, in the sense applicable to the professions of Surgery and Medicine.

A few eminent dentists, no doubt of extraordinary merit, enjoy a monopoly discouraging to the less fortunate members of their profession, in the limited spheres of aristocracy and wealth; but the great bulk of the community, whose patronage is essential to the extensive encouragement of competitors, take no interest in the profession of Dentistry, being without either means or incentives, such as I have described as peculiar to our country, for placing the profession on its proper footing, side by side with its co-ordinate professions.

Our own glorious country, gentlemen, is the legitimate country of progression;—conspicuous in this emphatic age of progression—when the onward cry of the moral and physical world is, "excelsior! excelsior!!"

Whether the first movement of discovery originates in the hardy regions of northern Europe, or in the enervating climate of half civilized Asia, the impulse which is to render it utilitarian and universal, is reserved for us. Destiny has assigned to us the faculty of capping every climax. Nature, God bless her! has bountifully bestowed upon the sons and daughters of Uncle Sam, busy brains of ceaseless energy, tough thews and sinews of extraordinary endurance, and flesh and blood unsurpassed in symmetrical proportions.

She has endowed us with her noblest forests, her most flowery and extensive prairies, her blandest climates, her largest and longest rivers, and the most prolific of populations; and, as to her gifts of thunder and lightning, we all know that we can't be beat.

Thus, whilst Nature has favored us with her choicest blessings, Art yields to us the palm in adaptation of her resources. Our Steamboat navigation, our Railroad go-ahead-itiveness, our Telegraphic extension of intercommunication, speak trumpet-tongued of our national vitality. Already is the old world becoming tributary to us for our giant locomotives, our printing presses of unrivaled speed, and our short-cuts to education and science. Let us, gentlemen, indulge the feasible and honorable ambition of rendering the same old world tributary to us for instruments, materials, and operative skill in our profession. Let us be stimulated by the conviction that her monarchies cannot rival us, because they have not the same combination of circumstances which we enjoy. That invaluable work, the American Journal of Dental Science, has rendered familiar to you, the labors of eminent men in our profession, during the last half century, both in the old and new worlds, to elevate it to its proper rank among the practical sciences in the field of utilitarianism.

It is apparent, in their investigations, that the effects of cooperation did not begin to be felt in this country till very recently. Associations of Dental Surgeons seem suddenly to have sprung up in the United States within the last ten or twelve years.

It appears that at first the promised benefits of these associations were not thought, by some, to be realized to the extent expected. But it appears to me that too much was expected, and that due consideration was not given to the nature of progress, and the necessity of allowing it time for its sure advance, step by step, in its steady march. I conceive that philosophical inquiry would establish the fact, that as much has been accomplished by association up to the present time, as could in the nature of things, be accomplished, under the circumstances. Those who revert disparagingly to the too easy admission of tyros in our formations of associations, do not reflect upon the two important facts, that, before there could be exclusion, the power and the right to exclude must pre-exist; and that cultivation, pruning, and proper training, might reasonably be expected, eventually, to remedy imperfections. A philanthropic mind would naturally conclude that a mere pretender who had imposed himself unworthily into a scientific association in its formation, would very soon find himself out of place, and that his self-pride would stimulate him to the acquisition of that skill and knowledge which would entitle him to the position he had assumed. And even, on the supposition, that some impostor might be so destitute of either principle or self-pride as to aim, not at all at improvement, but solely at imposition abroad by the exhibition of his name in an association, the cheat could not long exist; for the machinations of an individual would soon prove unavailing against the superior career of the truly scientific and skilful.

I think I may safely assume the position that, with regard to the admission of ignorant pretenders to our profession, in the formation of our societies, it would, in all probability, afford an appropriate opportunity of testing the very purpose of scientific association,—that of effecting such instruction, by pointing out sources of information, theoretical study and skilful expositions, as must improve and enlighten every member needing such professional light, until the whole profession should become elevated in public estimation; and I trust I shall not be denied the conclusion, that the ignoramus must indeed be wilfully and hopelessly incorrigible, who, with such advantages inviting him, would not speedily become an enlightened and acceptable member. It should not be forgotten that the object of professional association, is not to form a combination of self-elected exclusives who form their standard of perfection by the measure of their own attainments. Such men stand in no need of association for their own advancement, because they either are, or assume to be, above all competition. It is to those of less opportunity for scientific education and practical knowledge, that association with the more fortunate and eminent leaders of their profession, becomes most necessary and most desirable. And so far as the public at large is concerned, it is infinitely of more importance and benefit to the community, that scientific associations should be formed for the instruction and improvement of the comparatively ignorant in every profession, than for the mere conservatism of a professional oligarchy. I rejoice to say, that, however different may be opinions on the subject of the admission of members below a given grade of professional standing, the true principle of association amongst us, is thoroughly sustained. We find in all our associations of Dental Surgeons, the leading men of our profession nobly contributing the lights of science and skill which they themselves have attained, for the benefit and encouragement of their less fortunate brethren. They doubt not that by that means, the great object at which they aim, will be best and most speedily accomplished,—the elevation of their profession in the ranks of the useful and practical sciences, and in the estimation of the public at large. Far-seeing in their perceptions of a great truth to be revealed in the principle of association, they see the universal estimation in which professional men, who are known to be members of such scientific associations, must be held by an enlightened community, in contrast with self-lauded pretenders who have no real evidence to produce or refer to, in support of their professional assumptions.

Much has been said against the conduct of the public press in lending the aid of editorial influence to the impositions of quackery—to the detriment of regular practitioners. This of course is applicable to the medical as well as the dental profession. But, apart from the ethics of the question, I doubt if the conduct of the press, so much and so loudly exclaimed against, is, after all, worth contesting. If a profession of scientific men buckle on their armor and present themselves in platoon before a battery, whether of great or small guns, which they know to an absolute certainty, must be loaded with blank cartridges, they certainly would be inexcusable if they turned pale at every discharge, or threw themselves flat on their faces at every flash in the pan. But how much more inexcusable would they prove to be, at exhibiting such alarm, if they knew that the battery was not at all directed to them, but pointed the reverse way and was only firing a salute in honor of some mountebank clothed in shreds and patches, and universally laughed at by the discerning and enlightened? What is the true and well-known character of newspaper puffing? Is it not thoroughly understood? Is any one, of any consequence, deceived by it? It is a mere conventional jumble of words, paid for by the measure; known, when vended, to be worth nothing, but supposed to be foolishly esteemed most virtuous and effective by those whose imaginations are sufficiently lively to place faith in amulets or a Royal touch for the King's evil. We all know that editors of newspapers have two branches of editorial duties to perform; one is that of writing strong editorials for their papers, in which they pour out with a full tide of soul, all the truthfulness, earnestness and vehemence of their party sincerity; the other is, in the intervals of their paroxysms of editorial rage, to hold their pens merely, and let them run along inditing rapid common places of a business character for those who choose to pay their money for them, or contribute their patronage of advertising, to the business man of the establishment.

The language of these editorial puffs is perfectly innocent. The relation subsisting between the PUFFER and the PUFFED, is thoroughly understood. What has been said by a very amusing writer, of the Chinese, may be considered an apt illustration. "The most solemn expressions of respect and kindness," says this entertaining writer, "will pass between them almost upon no occasion. The greatest honor and esteem they will declare for one whom perhaps they never saw before; they will be entirely devoted to his service and interest without any reason; infinitely and entirely obliged for no favor, and extremely concerned for him, indeed afflicted for no cause." So, the quack, for a consideration, extracts from the editorial pen, the most solemn expressions of respect and kindness, on no occasion; the greatest honor and esteem for his quackery; an entire devotion to the service and interest of the community in recommendations without rhyme or reason; an acknowledgment of being infinitely and entirely obliged for the favor of being acquainted with the repository of so much genius; and great concern, if not affliction, that the world should fail to "put money in his purse."

The exertions of quackery are, I believe, less injurious to the professions than to the community. In a large mass of cases, the effect must be to bring under the treatment of scientific men, a greater number of cases than would come before them in the ordinary operation of natural causes of disease. And it appears to me, that the principles of our professional associations, which are so essentially inimical to the efforts of quackery, will, in due time, be appreciated by the community as its best safe-guard against the evils of quackery.

This is essentially a matter-of-fact age. The rising generation is not being trained up in the same leading strings of the children of our early day and generation. Instead of the story books of giants, phantoms and fairies with which our childish imaginations used to be instructed, the children of the present day are fed with intellectual aliment of historical or scientific truths and mathematical deductions. When children so trained advance to puberty, and step forth into the general walks of life, they will have no excitable imaginations to give credence to the professions of quacks or their laudations by newspaper puffs. They will probe pretensions to the quick, and trust only to evidences of truthfulness.

Here then we have two elements of irresistible power already sapping the foundation of quackery, and rendering sure its final overthrow:—the spread of scientific associations, and the seeds of truthfulness, germinating in an infant soil.

And it may not be inappropriate to remark, that of all the signs of the rapid approach of man to his final destiny,—the triumph of mind over matter,—the developments of progress in our day, are the most striking.

We cannot fail to perceive, that there is a culmination of the moral, physical and mental elements, connected with our state of being, which tends towards that illumination which must make all things clear that have been hitherto imperfectly seen, as through a mist, and render easy the path to human perfection and universal happiness. Doubtless, we, in our humble sphere, *are, here,*—by our efforts in association to reach that perfection which alone can complete our branch of the general stock of knowledge and science,—contributing our mite to the great work of human perfectibility.

How sublime is the contemplation of this progression! How chastening in its influences! It dictates to us a universal philanthropy, preparatory to our entry into the temple of universal peace. Fain would I, if I had the gift of eloquence, inculcate in appropriate terms, in closing my address, the true doctrine of happiness, that we best consult our own ease and enjoyments by contributing to that of others. Professionally, you have constant opportunities of relieving your fellow creatures from physical pain,—philanthropically let it also be your aim to sooth the mental pains of the afflicted, and cheer the faint-hearted, whenever you meet the weary *in your path* of life. Say to him, with the poet, whose inspiration comes from on high:

There is a voice within me,
And 'tis so sweet a voice,
That its soft lispings win me,
Till tears start to mine eyes.
Deep from my soul it springeth
Like hidden melody;
And ever more it singeth
This song of songs to me:—
"This world is full of beauty,
As other worlds above;
And if we did our duty,
It might be full of love."

For the leaf-tongues of the forest—
The flower-lips of the sod—
The birds that hymn their raptures
Into the ears of God—
And the sweet wind that bringeth
The music of the sea—

Have each a voice that singeth
This song of songs to me:—
"This world is full of beauty,
As other worlds above;
And if we did our duty,
It might be full of love."

